# Soil fungal community is more sensitive than bacterial community to modified materials application in saline–alkali land of Hetao Plain

**DOI:** 10.3389/fmicb.2024.1255536

**Published:** 2024-02-05

**Authors:** Xiaolong Bai, En Zhang, Jinmin Wu, Donghai Ma, Chaohui Zhang, Bangyan Zhang, Yunpeng Liu, Zhi Zhang, Feng Tian, Hui Zhao, Bin Wang

**Affiliations:** ^1^College of Agriculture, Ningxia University, Yinchuan, China; ^2^Tumote Right Banner Agricultural Technology Extension Center, Baotou, China

**Keywords:** saline–alkali soil, modified materials, bacteria, fungi, high-throughput sequencing

## Abstract

Soil salinization has become a major challenge that severely threatens crop growth and influences the productivity of agriculture. It is urgent to develop effective management measures to improve saline–alkali soil. Thus, in this study, soil properties, microbial communities, and function under desulfurization gypsum (DE), soil amendment (SA), farm manure (FA), and co-application of desulfurization gypsum, soil amendment, and farm manure (TA) in a field experiment were examined by high-throughput sequencing. The results showed that the application of modified materials is an effective approach in improving saline–alkali soil, especially TA treatment significantly increased the content of available phosphorus (AP), available potassium (AK), soil organic matter (SOM), and alkaline hydrolysis nitrogen (AHN) and decreased pH, bulk density (BD), and electrical conductivity (EC). The application of modified materials resulted in notable enhancement in fungal diversity and altered the composition and structure of the fungal community. Conversely, the effect on the bacterial community was comparatively minor, with changes limited to the structure of the community. Regarding the fungal community composition, Ascomycota, Mortierellomycota, and Basidiomycota emerged as the dominant phyla across all treatments. At each taxonomic level, the community composition exhibited significant variations in response to different modified materials, resulting in divergent soil quality. The TA treatment led to a decrease in Mortierellomycota and an increase in Ascomycota, potentially enhancing the ability to decompose organic matter and facilitate soil nutrient cycling. Additionally, the sensitivity of fungal biomarkers to modified materials surpassed that of the bacterial community. The impact of modified materials on soil microbial communities primarily stemmed from alterations in soil EC, AP, AK, and SOM. FUNGuild analysis indicated that the saprotroph trophic mode group was the dominant component, and the application of modified materials notably increased the symbiotroph group. PICRUSt analysis revealed that metabolism was the most prevalent functional module observed at pathway level 1. Overall, the application of modified materials led to a decrease in soil EC and an increase in nutrient levels, resulting in more significant alterations in the soil fungal community, but it did not dramatically change the soil bacterial community. Our study provides new insights into the application of modified materials in increasing soil nutrients and altering soil microbial communities and functions and provides a better approach for improving saline–alkali soil of Hetao Plain.

## 1 Introduction

Soil salinization has become a worldwide environmental challenge that significantly hampers crop growth and agricultural sustainability (Xu et al., 2018). Saline–alkali soils in China cover an estimated area of approximately 3.6 × 10^7^ ha, predominantly found in the desert and semi-desert regions of the northern, northeastern, and northwestern parts of the country (Mao et al., 2016). Saline–alkali soils are characterized by high salinity, degraded soil structures, nutrient deficiency, and diminished biological characteristics, leading to crop loss and decreasing crop yields (Xu et al., 2019; Yang et al., 2020). The development of strategies for the restoration of saline–alkali soils became crucial to ensuring sustainable crop productivity and promoting ecosystem development in China (Meena et al., 2016).

Soil microbes are crucial participants within the soil ecosystem, playing a pivotal role in mediating the material cycle and nutrient flow in the soil function and constituting a vital component of the soil health and quality evaluation system through processes, such as organic residue decomposition, enzyme release, and nutrient recycling (Saifullah Dahlawi et al., 2018). Excessive soil salinity and inadequate nutrient levels may negatively impact microbial growth and diversity. The application of modified materials to saline soil can mitigate the adverse effects of salinity on microbes and enhance the soil nutrient content to promote microbial growth and development (Bhaduri et al., 2016). Research on saline soils conducted to date has shown that microbial diversity and community exhibit high sensitivity to amendments (Huang et al., 2017; Zheng et al., 2018). Related studies have demonstrated the characteristics of microbial communities under various modified materials (Zhao et al., 2019; Liao et al., 2021; Guo L. et al., 2022; Mao et al., 2022), and the application of exogenous modified materials to saline–alkali soils has demonstrated efficacy in enhancing soil fertility and altering soil microbial community and biodiversity (Luo et al., 2018). A study by Chang et al. (2022) demonstrated that the application of organic amendments increases microbial diversity in saline–alkali soils and enriches microbial communities, particularly those species associated with soil aggregate stability (e.g., Ascomycetes).

Desulfurization gypsum (DE), a by-product of sintering flue gas desulfurization, has gained significant attention for its potential for environmental improvement (Zhao et al., 2018; Wang et al., 2021). Numerous studies have highlighted the significant advantages of utilizing desulfurization gypsum as a soil conditioner, such as reduction in nutrient leaching (He et al., 2019), improvement in soil fertility (Tao et al., 2019; Zhang et al., 2021), and promotion in plant growth and yield (Wang et al., 2023). A study by Shi et al. (2019) investigated the effect of applying gypsum on microbial communities, and the results indicated gypsum application led to changes in the structure and diversity of the bacterial community. Organic amendments, including farmyard manure, crop residue, and waste, have proven to be effective in improving the qualities of saline soils. The application of organic amendments in saline–alkaline soils could increase soil nutrient content, microbial biomass, biological abundance, and crop yields (Medina Litardo et al., 2022). For example, Guo L. L. et al. (2022) showed that applying organic amendments affected soil microbial community and diversity; specifically, applying crop residue and lignite humic acid significantly increased the abundance of Acidobacteria, while cow manure increased the proportion of Skermanella, Micrococcaceae, and Rubrobacter; and lignite enhanced the proportion of nitrogen cycle groups but decreased the microbial proportion of carbon cycle groups. A recent study by Zhang et al. (2022) revealed that long-term organic fertilizer application facilitates the proliferation of plant growth-promoting bacteria groups, such as Thiobacillus, Methylobacter, Rhodoferax, Lysobacter, and Flavobacterium, while the abundance of the halophilic genera was significantly decreased. Soil ameliorants, consisting of beneficial functional microbes (PGPR) along with a suitable substrate, are a specialized type of fertilizer that not only improve the soil physicochemical properties and enhance nutrient supply capacity but also alter the soil microbial community (Lu et al., 2020; Gu et al., 2022; Huang J. et al., 2023). Numerous studies have provided evidence that soil ameliorant application can serve as an effective strategy for improving soil properties (Naher et al., 2021), promoting soil microbial activities that are closely associated with nutrient cycling and disease resistance (Lu et al., 2020; Huang B. et al., 2023), and enhancing soil fertility in a saline–alkali ecosystem (Chen et al., 2021). These studies support the application of soil ameliorants as an effective agro-technological approach to address salt constraints. However, much less attention has been given to the influence of modified materials on the soil microbial community using molecular microbiology techniques for the remediation of saline–alkali soil in Hetao Plain, China.

We hypothesized that applying modified materials to saline–alkali soils could effectively reduce soil salinity, subsequently improve the soil physicochemical properties, and alter microbial communities, thus improving soil productivity. To test this hypothesis, a field experiment was carried out in a typical saline–alkali region. The aims of this study were as follows: (1) Modified materials can improve the physicochemical properties of saline–alkali soil, enhance soil nutrient content, and improve soil productivity; (2) the co-application of the modified materials would have a much evident effect on soil properties, microbial diversity, and composition compared to individual additions; and (3) the relationship can be explored among soil properties, microbial communities, and functions.

## 2 Materials and methods

### 2.1 Experimental field

The study area was located in Wayao Village, Meidaizhao Town, Tomote Right Banner, Baotou City, Inner Mongolia Autonomous Region, China (40° 14′–40° 51′ N and 110° 14′–111° 07′ E). This region is characterized by a typical continental semi-arid monsoon climate. The average annual precipitation was 346 mm, with a yearly average of 3,095 h of sunshine, the mean annual temperature was 7.5°C, and the frost-free period lasted for approximately 135 days. The initial soil physicochemical properties were pH of 8.42, electrical conductivity (EC) of 1.22 mS·cm^−1^, available phosphorus (AP) of 11.88 mg·kg^−1^, available potassium (AK) of 64.67 mg·kg^−1^, soil organic matter (SOM) of 13.55 g·kg^−1^, and alkaline hydrolysis nitrogen (AHN) of 63 mg·kg^−1^.

### 2.2 Experimental design and treatments

The experiment setup followed a randomized block design with three replications of plots (5 m × 6 m). The distance between the plots was maintained at 1 m. The experimental treatments consisted of the following: (1) non-treated control (CK), served as the control with no modified materials applied; (2) DE, involving the amendment of desulfurization gypsum at 15 t·hm^−2^; (3) SA, involving the amendment of soil amendment at 15 t·hm^−2^; (4) FA, involving the amendment of farm manure at 7.5 t·hm^−2^; and (5) TA, involving the amendment of the mixture of DE, SA, and FA, with the half amount as DE, SA, and FA treatments. Desulfurization gypsum was obtained from the Ningxia Maliantai coal mine, farm manure was obtained from a local centralized composting area, and soil amendment was provided by Fengyuan Biotechnology Co., Ltd., Ningxia. The composition of modified materials used in this study is shown in Table 1. Prior to maize planting, the modified materials were evenly sprinkled on the soil surface and mixed with the plow layer soil (0–20 cm) using a rotary cultivator to ensure uniform distribution. Irrigation was carried out for 24 h after the ameliorants were applied. The same method was applied to the CK treatment. Following the planting process, no additional field management practices, such as fertilization, were performed, except for watering. The maize cultivar used in this experiment was Hongfeng 556, which was planted after modified materials sank in the soil.

**Table 1:** Chief characteristics of the modified materials used in the study.

Modified materials			
Parameter	DE	SA	FA
pH	8.5	8	8.14
Water content (%)	10	12.15	28.36
OMC (%)	–	25	40
NPK content (%)	0.1	5	4
MS content (billion/g)	–	2	–
Others	–	Furfural residue, calcium ammonium nitrate	–

OMC, organic matter content; MS content, microorganism content.

### 2.3 Sample collection and analysis

Soil samples were collected when the maize was harvested. A five-point sampling method was employed to gather samples from each plot. In total, 15 soil samples (5 treatments × 3 replicate) were obtained. At each sample point, the collected samples were divided into two portions: One portion was subjected to a process of natural air-drying, homogenization, and sieving (< 2 mm) to remove any residues of silica sand or plant roots to analyze the soil physicochemical properties, and the other portion was immediately placed in an ice box, transported to the laboratory within 24 h, and frozen at −80°C for high-throughput sequencing.

### 2.4 Measurement of soil physiochemical properties

Soil pH was measured using the potentiometric method (soil/water = 1:5). Soil EC was determined using the DDS-11A conductivity meter (soil/water = 1:5). Available phosphorus (AP) was measured using the NaHCO_3_-extracted method. Available potassium (AK) was determined using the NH_4_OAc-extracted method. The soil organic matter (SOM) was measured using the potassium dichromate oxidation-capacity method. Alkaline hydrolysis nitrogen (AHN) was analyzed using the alkali solution diffusion method.

### 2.5 DNA extraction and high-throughput MiSeq sequencing

Total DNA extraction was performed using the TGuide S96 Magnetic Soil/Stool DNA Kit (Tiangen Biotech (Beijing) Co., Ltd., China). To determine extracted DNA concentration and purity, the qubit method (Quant-iTTM ds DNA HS Reagent and HS Buffer) was employed, and 1.8% agarose gel electrophoresis was performed to assess the DNA quality. To determine the composition and diversity of bacterial and fungal communities under different modified material treatments, 16S rRNA and ITS amplicon sequencing technologies were utilized. The bacterial 16S rRNA gene V3-V4 region was amplified using primers 338F (5′-ACTCCTACGGGAGGCAGCA-3′) and 806R (5′-GGACTACHVGGGTWTCTAAT-3′) (Bulgarelli et al., 2012; Lundberg et al., 2012). The fungal ITS gene was amplified using primers ITS1F (5’-CTTGGTCATTTAGAGGAAGTAA-3′) and ITS2 (5’-GCTGCGTTCTTCATCGATGC-3′). The amplification conditions were as follows: predenaturation at 95°C for 5 min; 25 cycles (denaturation at 95°C for 30 s, annealing at 50°C for 30 s, extension at 72°C for 40 s), and a final extension at 72°C for 7 min. The PCR amplification of the 16S rRNA/ITS genes and the subsequent purification of the products were conducted according to the methods described by Wu et al. (2023). After the PCR analysis, the purified amplicons were then pooled in equimolar concentrations, and eligible libraries were performed using the Illumina NovaSeq sequencing platform at Beijing Bemac Biotechnology Co., Ltd.

### 2.6 Bioinformatics analysis

The raw sequence data obtained from high-throughput sequencing were filtered, spliced, and chimeras removed using QIIME2 software; operational taxonomic units (OTUs) were delineated at the 97% level of similarity; species were annotated and analyzed for abundance; and shared and unique OTUs between groups were analyzed using Venn diagrams. The raw sequences are available through the NCBI Sequence Read Archive (Accession No. SRP471127).

### 2.7 Statistical analysis

SPSS version 25.0 was utilized for conducting a one-way analysis of variance (ANOVA). All analyses, including α-diversity, community composition, β-diversity, linear discriminant analysis (LEFSe), and functional analysis, were conducted on the Biomarker Cloud Platform available at www.biocloud.net. Rarefaction curves were constructed utilizing Mothur at a 97% identity level, using the observed species richness as a basis. The analyses of FUNGuild and PICRUSt were performed using the R package “microeco” (v.0.11.0). Non-parametric tests and linear discriminant analysis were employed for biomarker discovery. Redundancy discriminant analysis (RDA) was performed in Bioincloud (bioincloud.tech/) to analyze the relationship between the microbial community composition and soil physicochemical properties. The “corrplot” package in R (version 3.1.2) was used to generate a heatmap illustrating the microbial family in relation to soil properties.

## 3 Results

### 3.1 Soil physical and chemical properties

Table 2 presents the soil physicochemical properties under modified materials. Compared to CK, modified materials enhanced the content of AP, AK SOM, and AHN. The AP and AK levels showed the highest in TA treatment, followed by FA and SA treatments, and DE treatment showed no significant increase compared to the CK treatment. The SOM content was significantly higher in the TA and FA treatments than in the CK treatment (*p* < 0.01), while there was no significant difference between the SA, DE, and CK treatments (*p* > 0.05). Additionally, only TA treatment led to a marked enhancement in AHN (*p* < 0.05). Soil bulk density (BD) and EC after DE, SA, FA, and DE + SA + FA application were 0.75%–5.22% and 40%–59.13% lower, respectively, than those of CK. Furthermore, compared to the CK treatment with a pH value of 8.27, DE and TA treatments showed a notable decline in pH with values of 7.87 and 7.77, respectively (*p* < 0.05).

**Table 2:** Soil physicochemical properties under modified materials.

Treatment	pH	BD (g·cm^−3^)	EC (mS·cm^−1^)	AP (mg·kg^−1^)	AK (mg·kg^−1^)	SOM (g·kg^−1^)	AHN (mg·kg^−1^)
CK	8.27 ± 0.10a	1.34 ± 0.01a	1.15 ± 0.12a	10.25 ± 0.50d	60.77 ± 2.39d	13.67 ± 0.35c	60.77 ± 1.17b
DE	7.87 ± 0.03b	1.29 ± 0.01b	0.51 ± 0.08bc	12.06 ± 0.87d	60.98 ± 1.92d	14.18 ± 0.26c	62.33 ± 1.48b
SA	7.92 ± 0.19ab	1.32 ± 0.02ab	0.69 ± 0.02b	14.83 ± 1.25c	72.62 ± 2.84c	15.28 ± 0.24c	63.25 ± 2.52ab
FA	7.95 ± 0.03ab	1.33 ± 0.01a	0.48 ± 0.09bc	20.52 ± 0.62b	82.59 ± 2.61b	18.65 ± 0.73b	65.33 ± 2.59ab
TA	7.77 ± 0.11b	1.27 ± 0.02b	0.47 ± 0.03c	25.85 ± 1.31a	92.56 ± 1.84a	20.65 ± 0.48a	70.00 ± 2.02a

CK, non-treated control; DE, desulfurization gypsum; SA, soil ameliorant; FA, farm manure; TA, mixed treatment. Different letters in the same column indicate significant differences (*p* < 0.05).

### 3.2 Sequencing results and microbial community diversity

**Figure 1:**
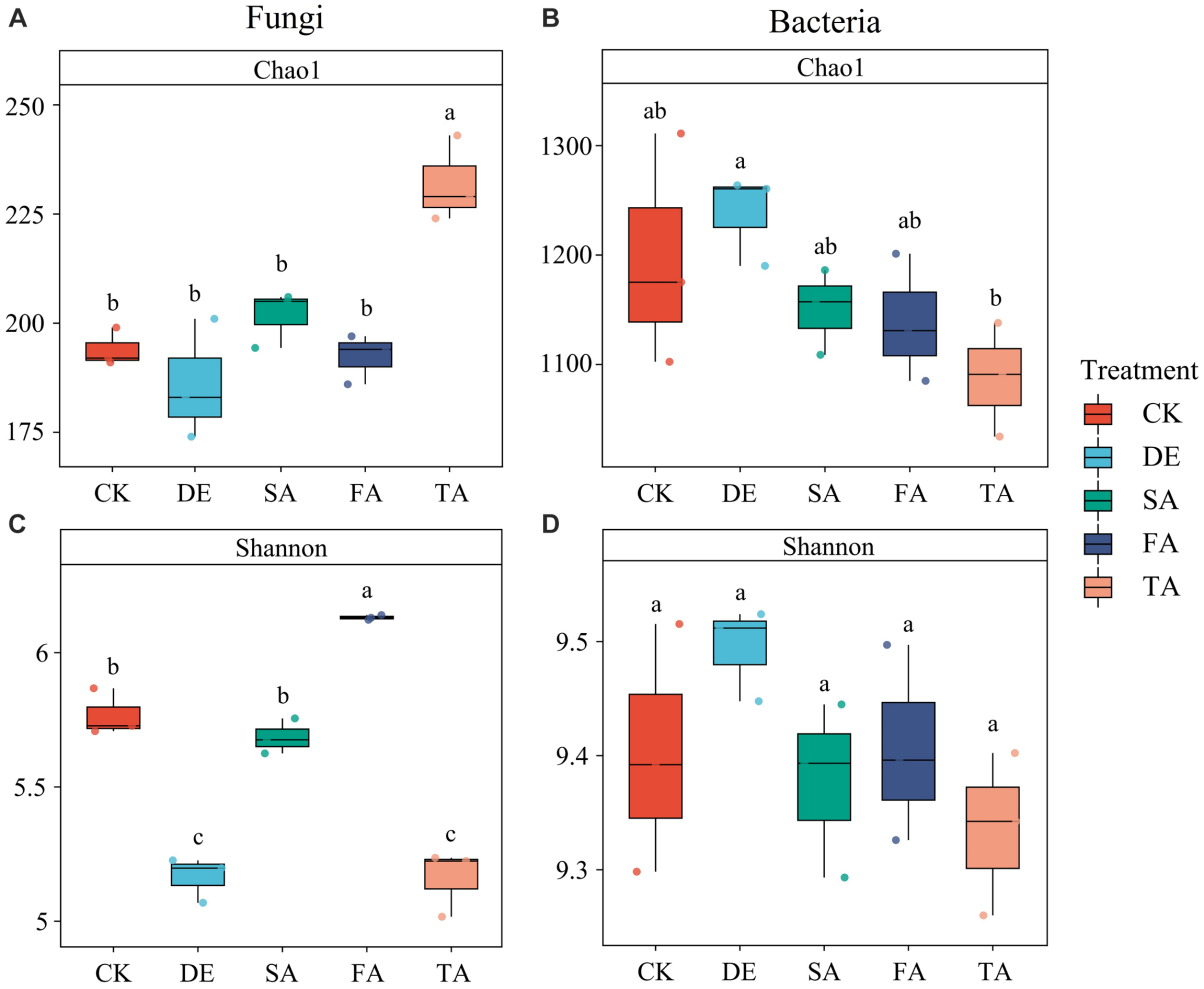
Soil microbial α-diversity under different modified materials. **(A)** Chao1 index of soil fungi; **(B)** Chao1 index of soil bacteria; **(C)** Shannon index of soil fungi; **(D)** Shannon index of soil bacteria. Note: CK: non-treated control; DE: desulfurization gypsum; SA: soil ameliorant; FA: farm manure; TA: mixed treatment.

After quality filtering and clustering of OTUs, the rarefaction curves of 15 samples displayed a saturation plateau (Supplementary Figure S1), indicating that the data were adequate for capturing a substantial portion of the microbial diversity present in the soil. The species diversity (Shannon) and richness (Chao1) of the fungal and bacterial communities were examined across various modified treatments (Figure 1). The results revealed that the fungal community exhibited lower diversity in DE and TA treatments than in the CK treatment. In contrast, it was observed that the TA treatment led to an increase in species richness in the fungal community. For bacteria, SA, FA, and DE + SA + FA application decreased the Shannon and Chao1 indexes, while DE led to an increase in species richness and diversity, though there was no significant difference between modified and control treatments.

**Figure 2:**
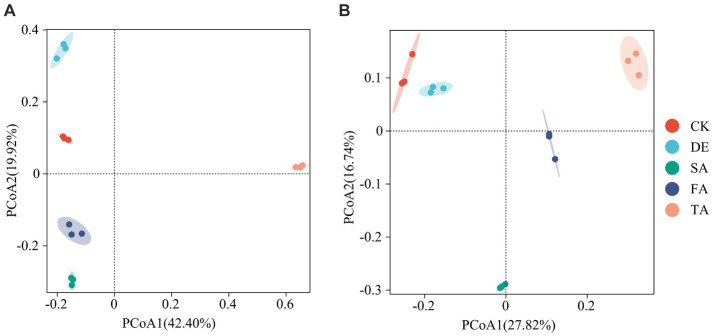
PCoA of the fungal **(A)** and bacterial **(B)** communities under different modified materials.

Principal coordinate analysis (PCoA) was conducted to determine the structure of soil fungal and bacterial communities across various treatments based on OTU taxonomy. The PCoA1 and PCoA2 principal components accounted for 42.4% and 19.9% of the species variation in the fungal community and 27.8% and 16.7% of the species variation in the bacterial community, respectively (Figure 2). Notably, the PCoA revealed that the TA treatment exhibited a distinct separation from the other treatments, suggesting that the TA treatment has a significant difference in the fungal community structure compared to the other treatments. Samples of the CK, DE, SA, and FA treatments clustered together, as evidenced by the clustering tree (Supplementary Figure S2).

### 3.3 Microbial community composition

**Figure 3:**
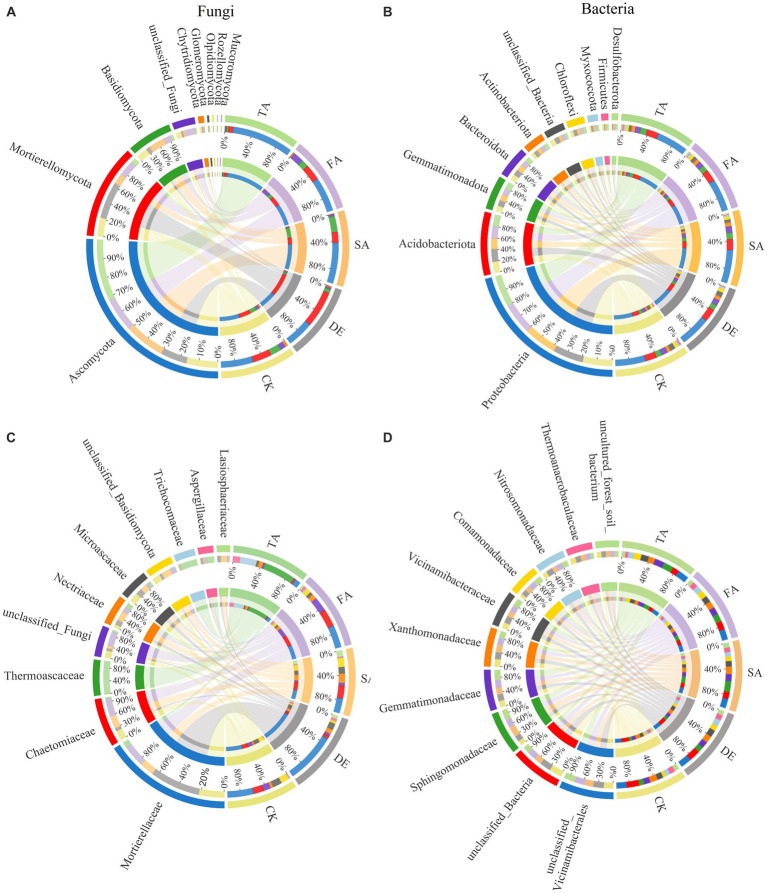
Relative abundance of fungal phylum **(A)** and family **(C)**, and bacterial phylum **(B)** and family **(D)** under different modified materials.

Variations in the fungal composition among different treatments were evident. Ascomycota, Mortierellomycota, and Basidiomycota were found to dominate the fungal community composition in this study (Figure 3A). The DE treatment significantly enhanced the relative abundance of Mortierellomycota but led to adverse effects on Ascomycota and Basidiomycota when compared to the CK treatment. Ascomycota are known for the decomposition of organic matter and promotion in soil nutrient cycling, and the abundance of Ascomycota is 45.69% in the CK treatment. Interestingly, the relative abundance of Ascomycota exhibited an increase of 21.07%, 6.53%, and 48.04% in the SA, FA, and TA treatments, respectively, compared to the CK treatment. The abundance of Basidiomycota showed a sharp increase under the SA treatment when compared to the DE, FA, and TA treatments. Moreover, the application of modified materials resulted in a significant decrease in the relative abundance of Chytridiomycota. Furthermore, at the family level, the relative abundances of Mortierellaceae exhibited a significant increase in the DE treatment while decreasing in the SA, FA, and TA treatments compared to the CK treatment. After applying modified materials, the relative abundance of Chaetomiaceae significantly increased in the SA and FA treatments by 20.04% and 43.24%, respectively, compared to the CK treatment. Conversely, compared to the control, the relative abundance of Microascaceae exhibited a decrease of 30.81%, 29.39%, 68.60%, and 76.36% in the DE, SA, FA, and TA treatments, respectively (Figure 3B). The bacterial communities were predominantly composed of Proteobacteria, Acidobacteria, Gemmatimonadetes, Bacteroidetes, and Actinobacteria (Figure 3C), which collectively accounted for more than 81.56% of the bacterial composition across all samples. The proportion of Proteobacteria increased by 1.62%, 9.84%, 6.54%, and 4.08% in the DE, SA, FA, and TA treatments, respectively, in comparison with the CK treatment. The relative abundance of Gemmatimonadetes and Bacteroidetes was highest in CK soil, while Acidobacteria exhibited the highest proportion in the DE treatment followed by the CK, SA, FA, and TA treatments. Moreover, at the family level, the application of modified materials increased the relative abundance of Sphingomonadaceae, with the SA treatment being the highest, followed by TA, FA, and DE treatments (Figure 3D). After applying the modified materials, the relative abundances of Gemmatimonadaceae and Comamonadaceae increased, while the relative abundances of Comamonadaceae and Comamonadaceae decreased compared to the CK treatment.

**Figure 4:**
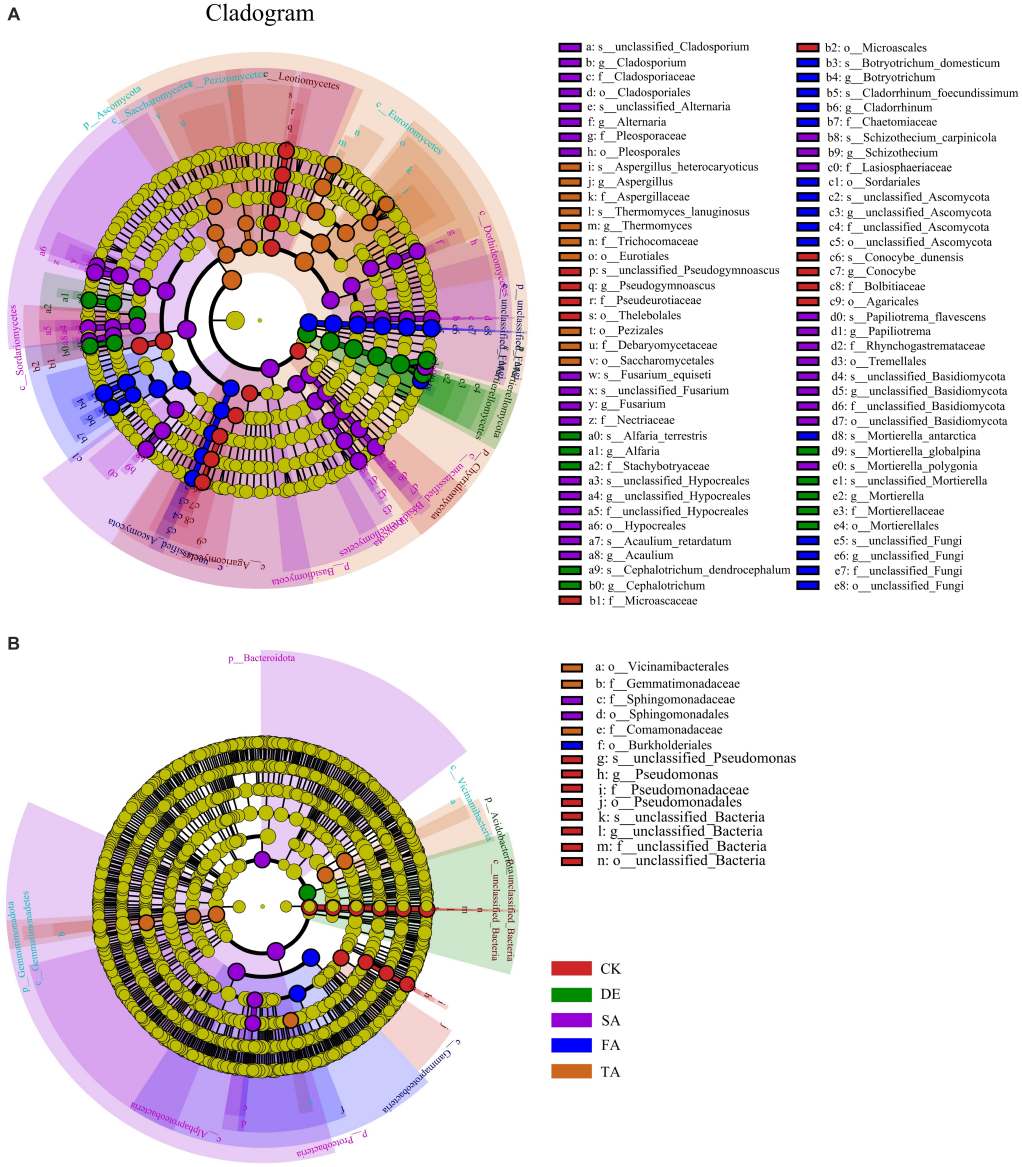
LEfSe results of fungal **(A)** and bacterial **(B)** biomarkers (from phylum level to genus level) sensitive to different modified materials. There are six circular rings in the cladogram (LDA > 4), and each ring accommodates all taxa within a specific taxonomic level, the arrangement of the circular rings from inside to outside represents the phylum, class, order, family, and genus, respectively. Each node situated on the circular ring represents a taxon affiliated with the respective taxonomic level. The nodes are differentiated by color to denote microbial populations that are significantly enriched in different treatments and have a notable effect on differences between groups; the dark yellow nodes represent microbial populations that are not significantly different across groups.

To better distinguish the variations of microbial species in response to modified materials and identify potential key microbes, we conducted the LEfSe (LDA > 4, *p* < 0.05) analysis to determine the microbial community (Figure 4). With respect to the fungal community, we identified 61 biomarkers across five phylum levels. Specifically, Chytridiomycota was the key biomarker in the CK treatment, while Mortierellomycota was the key biomarker in the DE treatment. The FA treatment enriched unclassified_Fungi, Basidiomycota, and Ascomycota as the key biomarkers in the SA and TA treatments, respectively. As for the bacterial community, fewer key biomarkers were observed compared to the fungal community, with only 12 biomarkers identified in all five treatments.

The Venn plots provided a visual representation of the similarity and specificity of the OTU composition under modified materials treatments, showing the number of shared and unique OTUs among different samples (Supplementary Figure S5). The comparison for fungi revealed that there were more shared OTUs between the CK and TA treatments than other treatments. Specifically, 57 OTUs were identified as common OTUs and the TA treatment had a higher number of unique fungal OTUs. In contrast to our findings with the fungal communities, the treatments with more shared OTUs were observed between the CK and DE treatments rather than other treatments, 331 OTUs were identified as common OTUs, and the CK treatment contained many unique bacterial OTUs.

### 3.4 Function of microbial community

**Figure 5:**
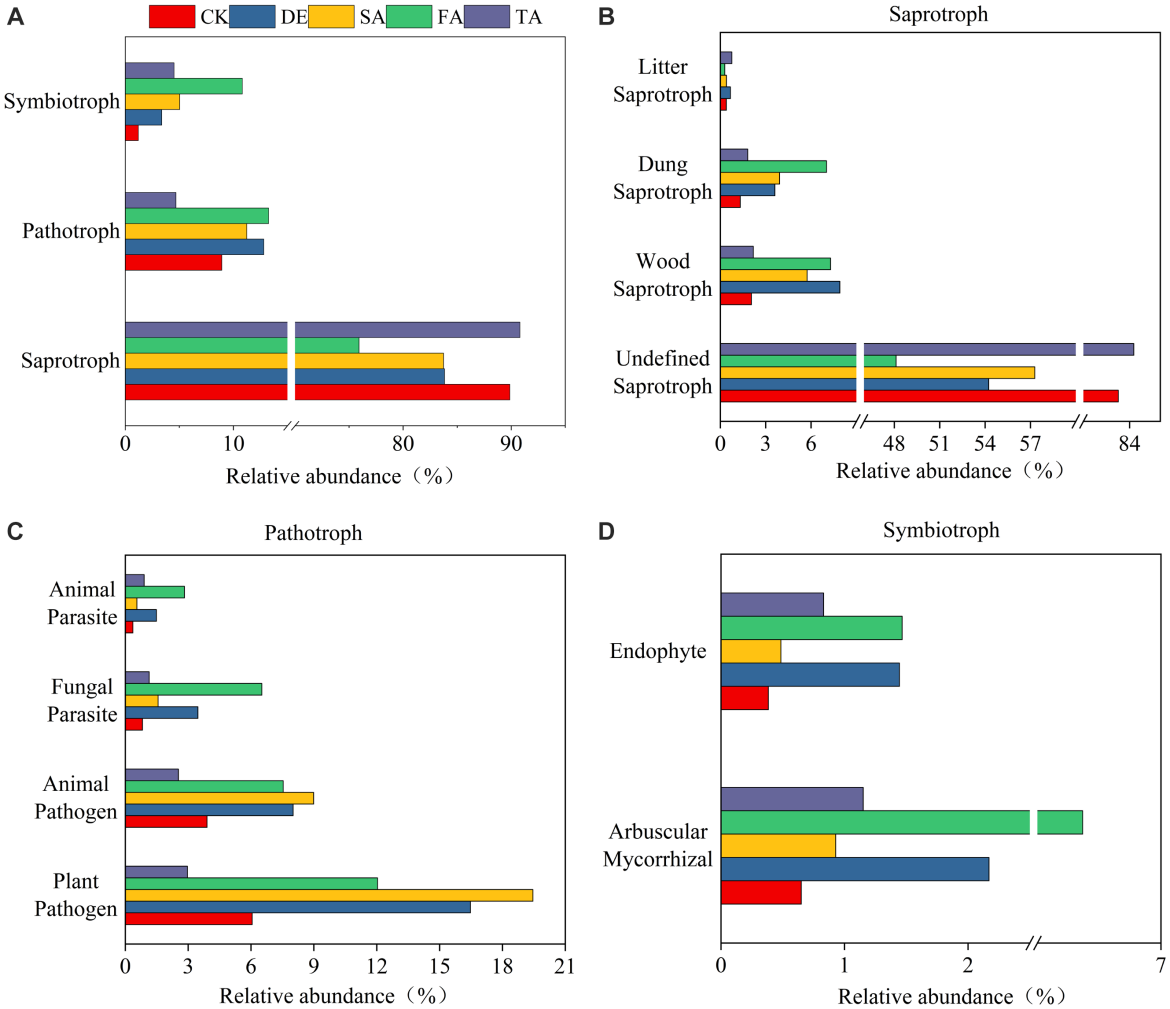
Fungal functional predictions under modified materials annotated by FUNGuild. **(A)** Functional groups of fungi; **(B)** Analysis of the saprotroph under modified materials; **(C)** Analysis of the pathotroph under modified materials; **(D)** Analysis of the symbiotroph under modified materials.

To explore how modified material affects the ecosystem community function, we predicted the potential ecological function based on ITS rRNA gene sequences using FUNGuild and 16S rRNA gene sequences using PICRUSt2. The results showed that the fungal community function was more sensitive to modified materials than the bacterial community function. Fungal communities could be classified into three trophic mode groups, with saprotroph being the significant component, comprising 75.92%–90.81% of the observed fungal OTUs (Figure 5A). The analysis of saprotroph composition indicated that the Wood saprotrophs in the DE, SA, and FA treatments were higher at 7.91%, 7.29%, and 5.74%, respectively, in comparison with the CK treatment (2.06%) (Figure 5B). Furthermore, the DE, FA, SA, and TA treatments exhibited a more significant presence of Dung saprotrophs when compared to the CK treatment. It was worth noting that the proportion of plant pathogen was higher than animal pathogen in pathogen group (Figure 5C). Among the symbiotrophic organisms, arbuscular mycorrhizal and endophytes were the primary groups. The relative abundance of arbuscular mycorrhizal fungi in the SA treatment was significantly higher at 6.37% in comparison with the CK treatment (0.65%) (Figure 5D). However, the bacterial functional groups are relatively stable among different modified materials (Supplementary Figure S6). The most predominant functional module observed at pathway level 1 was metabolism, and genes related to global and overview maps were found to be the prominent metabolic pathways at the level 2 KEGG ortholog function predictions.

### 3.5 Correlations between soil physicochemical properties and microbial communities

**Figure 6:**
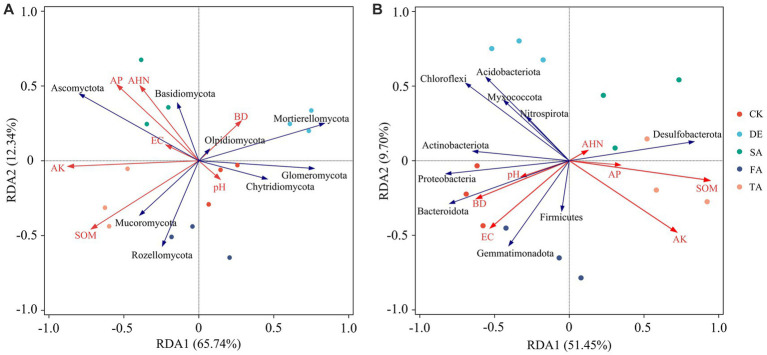
Redundancy analysis (RDA) of soil fungal **(A)** and bacterial communities **(B)** and environmental factors at the phylum level.

We performed an RDA to investigate the relationship between environmental parameters and soil microbial communities (Figure 6). The results demonstrated that soil physicochemical properties explained 78.08% of the variation in the fungal community and 61.15% of the variation in the bacterial community. Notably, the microbial communities in the CK soil exhibited a significant association with soil pH, BD, and EC, while the modified treatments displayed a positive correlation with AP, AK, SOM, and AHN. A higher abundance of Ascomycota found in modified materials was positively correlated with AP, AK, SOM, and AHN, while Mortierellomycota showed a negative relationship with pH and BD. Additionally, the main bacterial phyla Proteobacteria, Acidobacteria, and Gemmatimonadetes showed a positive correlation with soil pH, BD, and EC, but a negative relationship with soil nutrient parameters, AP, AK, SOM, and AHN. Interestingly, among the seven test soil indexes, the soil pH showed little effect in regulating soil microbial composition, while soil nutrients (AP, AK, SOM, and AHN) and EC had a stronger effect in driving the composition of the microbial community.

**Figure 7:**
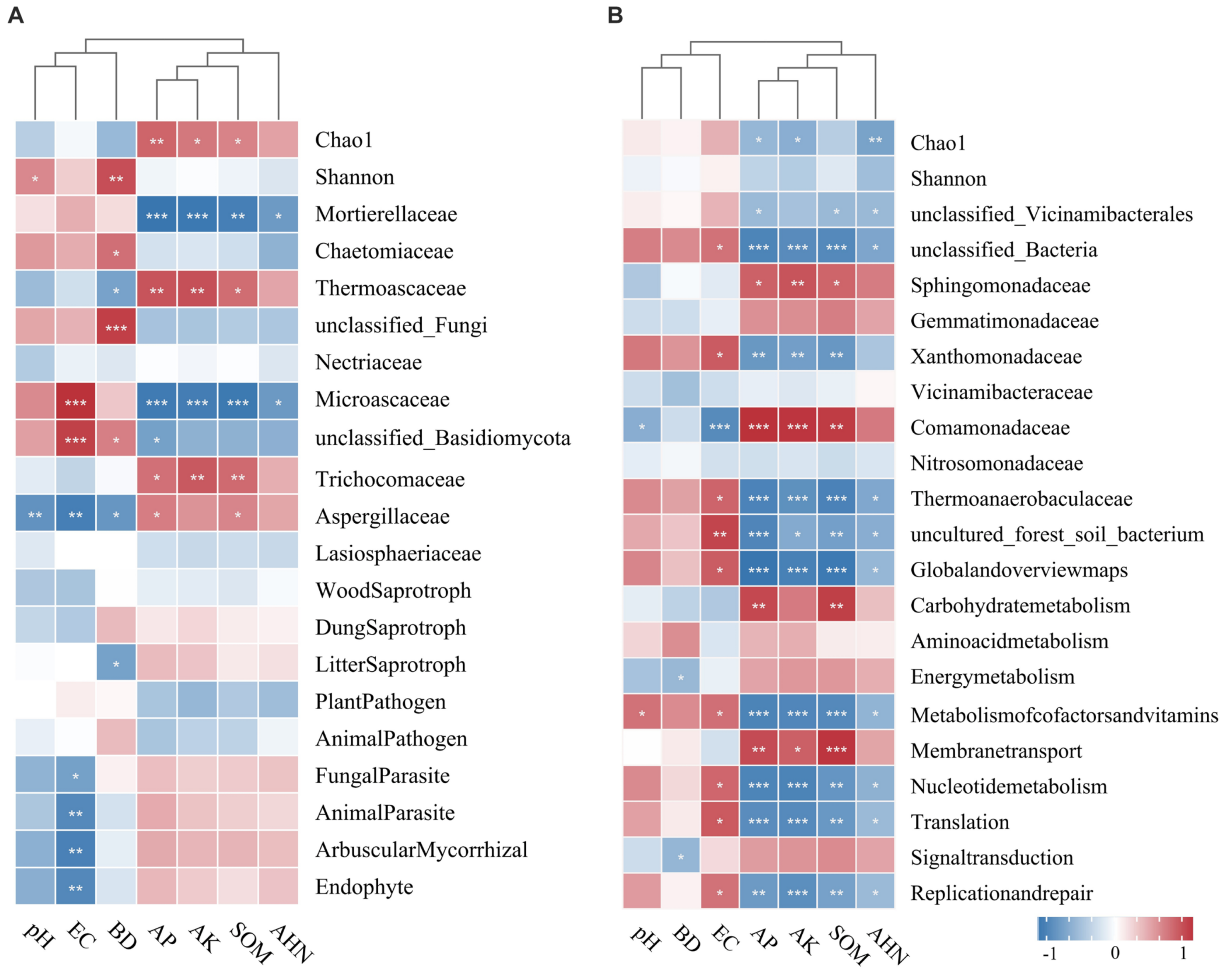
Phylogenetic clustering heatmaps visualizing the correlation between soil physicochemical properties and microbial diversity and the relative abundance of the top 20 main fungal **(A)** and bacterial **(B)** families. The correlation analysis was performed using Spearman’s method. *p < 0.05; **p < 0.01; ***p < 0 0.001.

The relationships among microbial diversity, dominant family, functional groups, and soil properties were examined using Spearman’s correlation analysis (Figure 7). The Chao1 index of fungi was significantly positively correlated with AP, AK, and SOM, and the Shannon index was positively correlated with pH and BD. AP, AK, and SOM had a positive relationship with the families Thermoascaceae, Trichocomaceae, and Aspergillaceae, while they had a negative correlation with Mortierellaceae and Microascaceae. Fungal parasites, animal parasites, arbuscular mycorrhizae, and endophytes were negatively correlated with EC, while litter saprotroph had a negative correlation with BD. The bacterial Chao1 index was negatively influenced by AP, AK, and AHN. Furthermore, AP, AK, and SOM had a positive influence on families Sphingomonadaceae and Comamonadaceae, but a negative influence on Xanthomonadaceae and Thermoanaerobaculaceae. The potential functional groups of bacteria were found to be influenced by EC, AP, AK, and SOM (*p* < 0.05).

## 4 Discussion

Salinity affects the soil microbial communities by causing soil structure deterioration, nutrient deficiency, and degradation of biological characteristics. The application of modified materials improved the soil structure and increased the content of nutrients (Fageria, 2012). In this study, desulfurization gypsum, soil ameliorant, and farm manure were applied to saline–alkali soil to investigate their potential effects on soil physicochemical properties and microbial communities (Leogrande and Vitti, 2019).

The field experiments indicated that soil parameters were differentially affected by modified materials. Desulfurization gypsum decreased the soil EC value, which can be attributed to its ability to release Ca^2+^ into the soil and replace exchangeable Na^+^ with Ca^2+^, forming Na_2_SO_4_ neutral salt (Yang et al., 2018). A study by Xie et al. (2017) observed that the unique habitat of saline–alkali soil in the Yellow River Delta, characterized by high salinity, inhibits microbial activity, and high Na^+^ levels also disrupt the structure of soil aggregates, thereby diminishing the effective sequestration and transformation of external carbon. Additionally, the increased presence of Ca^2+^ effectively reduces the concentrations of carbonate and bicarbonate, resulting in lower soil pH values (Table 2) (Zhao et al., 2021). Our study revealed that the application of modified materials led to an increase in soil nutrients, with the FA treatment giving better results than the DE treatment, consistent with the result by Shi et al. (2019). Desulfurization gypsum contained minimal amounts of N, P, and K, resulting in negligible release of associated nutrients into the soil (Kun and Xiaoping, 2019). Although C was retained in desulfurization gypsum, it primarily existed in the form of CaCO_3_ rather than organic matter. Applying farm manure is a conventional approach for improving salinized soil, which is beneficial for promoting the stabilization of soil agglomerates. The formation and stabilization of soil aggregates play a vital role in enhancing the soil structure, facilitating salt leaching, minimizing surface evaporation, and inhibiting salt accumulation in the topsoil (Meng et al., 2019). Soil ameliorants, comprising substrates and specific microorganisms with distinctive functions, have demonstrated environmental friendliness and positive impacts on soil conditions (Li et al., 2021). Earlier research also indicated that utilizing soil ameliorants enhanced the availability of soil nutrients while reducing soil EC and the concentrations of Na^+^ (Zhu et al., 2021). A comprehensive analysis of the three modified materials used to ameliorate saline soil revealed that DE outperformed SA and FA in reducing soil salinity, bulk density, and pH. Conversely, FA and SA showed advantages in enhancing soil nutrient levels compared to DE. Additionally, compared to a single application of modified materials, co-application of DE, SA, and FA performed better in both alleviating soil salinity and improving soil fertility (Table 2). These findings demonstrated that the simultaneous application of modified materials was a more effective approach for reclaiming saline soil.

The utilization of modified materials has proven effective in improving soil environmental conditions, thus directly affecting microbial diversity and altering the structure of soil microbial communities. Our findings revealed significant variations in soil fungal diversity under different simulated modified materials; in contrast, the same was not observed in the bacterial diversity, consistent with previous findings (Li et al., 2023; Zhang et al., 2023). In this study, we observed a significant increase in fungal diversity in response to the application of farm manure. This may be attributed to the presence of complex organic compounds in farm manure, which require various microorganisms for degradation (Ma et al., 2018). The results of the study support the previous findings that organic modified amendments consistently lead to higher microbial diversity (Esperschütz et al., 2007). Additionally, the fungi alpha diversity of DE and TA treatments exhibited the lowest, whereas the TA treatment showed the highest richness (Figure 1). The reason for the phenomenon may be as follows. Desulfurization gypsum can reduce salinity and improve the soil structure (Liu et al., 2023), which provides a favorable living environment for fungi, thereby intensifying the competition among fungal groups, and the growth of certain fungal groups may be inhibited or may even disappear, thus leading to a decrease in fungal diversity. Additionally, the combined application of modified materials, especially farm manure, provides microbes with a diverse range of unstable organic components in the soil (Wu et al., 2018), leading to a “preference” effect for certain specific fungi groups, thereby inhibiting the growth of other fungal groups and ultimately reducing fungal diversity. In addition, the positive relationship between the Chao1 index and AP, AK, SOM, and AHN suggested that the increased fungal richness is likely a result of improved nutrient levels in saline soil through the co-application of modified materials. Confirming our findings, a global meta-analysis conducted by Zhou et al. (2020) found that pH was the primary driver of α-diversity. This fact could be explained by the sensitivity of fungi to a pH change. Additionally, Spearman’s correlation analysis indicated a significant relationship between soil BD and fungal diversity (Jiang et al., 2019) (Figure 7A), suggesting that the application of modified materials reduced the bulk density and created a favorable habitat for fungi. However, the diversity of the soil bacterial community was not sensitive to the application of modified materials.

Soil microorganisms play a vital role in terrestrial ecosystems, with their diversity and community composition serving as indicators of both biotransformation efficiency and soil fertility (Manasa et al., 2020; Moreira et al., 2020). Our study demonstrated that modified materials had a stronger impact on the composition of the fungal community compared to the bacterial community (Figure 3). Ascomycota, Mortierellomycota, and Basidiomycota were the dominant fungal phyla in our study, corresponding with the results of Bei et al. (2018). Ascomycota, a type of saprophytic fungi (Guo et al., 2018), are known for their efficient decomposition of organic matter and their role in promoting soil nutrient cycling (Lin et al., 2019). Notably, the TA treatment resulted in a significant increase in the abundance of Ascomycota compared to the CK treatment and other modified treatments. Ascomycota and Basidiomycota play crucial roles as decomposers in the carbon cycle, utilizing their ability to secrete digestive enzymes to break down organic substances into smaller molecules. In agricultural soils, Ascomycota members are primarily responsible for decomposition. Previous studies have indicated a competitive relationship between Ascomycota and Basidiomycota, resulting in a negative correlation in their relative abundances (Ye et al., 2020), which can be clearly seen in the TA treatment of our study (Figure 3A). Basidiomycota, on the other hand, can be categorized as K-strategists as they appear later in the degradation stage and specialize in breaking down recalcitrant organic matter (Liu et al., 2023). The abundance of Basidiomycota decreased following the application of modified treatments involving DE or FA. A significant positive correlation was observed between Ascomycota and AP (*p* < 0.05), while an extremely significant negative association was observed between Basidiomycota and AK, suggesting that as nutrient levels increased, fewer nutrient resources were allocated to Basidiomycota. The application of desulfurization gypsum led to a decrease in the relative abundance of Ascomycota and an increase in the relative abundance of Mortierellomycota compared to the control. The divergent results regarding Ascomycota and Mortierellomycota in the DE treatment suggest that Mortierellomycota may not prefer nutrient-enrich environments and have a lower competitive advantage in soils with high nutrient content, which can be further illustrated by the negative correlation observed between Mortierellomycota and SOM and AK (Figure 6A). Additionally, modified material inputs resulted in lower abundances of Chytridiomycota and Glomeromycota. Despite their important roles in the decomposition of easily decomposable plant debris (Wang et al., 2020), the well-known arbuscular mycorrhizal symbiosis was exhibited by Glomeromycota with host plants (Choi et al., 2018). Their lower relative abundances following the introduction of modified materials might suggest the dominance and competitive ability of Ascomycota and Basidiomycota in such soils. At the family level, the application of modified materials increased the abundance of Mortierellaceae, Chaetomiaceae, and Nectriaceae, indicating an enhancement in the growth and development of beneficial salt-tolerant fungi in salinized soil. Mortierellaceae, known for its acid production ability, exhibited an extremely significant negative correlation with AK and AP in this study (*p* < 0.001), confirming that this microorganism might not thrive in environments with high nutrient content and was more abundant under the DE application compared to the control, potentially contributing to a lowered pH value in plots with DE application (Li et al., 2015). Consistent with previous studies, SOM played a vital role in shaping the fungal community composition based on RDA and Spearman’s correlation analysis. As documented by Ullah et al. (2019), the majority of fungi are heterotrophs and rely on exogenous carbon sources for growth, resulting in a significant influence of labile SOM on their abundance.

As for bacteria, the dominant bacterial phyla identified in this study were Proteobacteria, Acidobacteria, Chloroflexi, Firmicutes, Bacteroidetes, and Actinobacteria, which are commonly observed in similar ecosystems (Liang et al., 2021). Contrary to the fungal community composition, the variation in soil physicochemical properties induced by modified materials did not significantly affect the bacterial community composition. There was a positive correlation between Proteobacteria and BD, implying a preference for this microorganism for colonizing loose environments. However, Sphingomonadaceae, belonging to Proteobacteria, exhibited a positive correlation with AP, AK, and SOM (Figure 7). Previous studies have reported the involvement of Sphingomonadaceae in various soil processes, including carbon cycling, nitrogen fixation, pathogen inhibition, and promotion of plant growth (Stone et al., 2021). Additionally, a strong negative correlation between Chloroflexi and SOM suggests a potential lack of competitiveness of this microorganism in such ecosystems, resulting in lower abundance in this study (Ren et al., 2021).

FUNGuild is a widely utilized database for comparing fungal functions and conducting specific functional classifications of fungi (Nguyen et al., 2016; Mao et al., 2020). In this study, we observed that the fungi functional groups categorized by FUNGuild exhibited differential responses to various modified materials, with saprophytic fungi emerging as the dominant functional guilds, aligning with the findings of Ning et al. (2021). One possible explanation is that saprophytic fungal is closely linked with the breakdown of organic matter (Brabcová et al., 2018) and plays a critical role as soil decomposers (Eichlerová and Baldrian, 2020). Additionally, our study identified a notable increase in the mycorrhizal function in the DE and FA treatments compared to the control. Arbuscular mycorrhizal fungi (AMF) are known for their ability to enhance soil salinity tolerance in plants (Wang et al., 2018; Chang et al., 2023). Research has demonstrated that AMF can symbiotically associate with approximately 80% of land plants, boosting root system absorption and nutrient supply for plant growth (Berruti et al., 2016). The application of soil ameliorant leads to the proliferation of N-fixing bacteria. Numerous studies have demonstrated the vital role of N-fixing bacteria and AMF in supporting plant growth, promoting soil nutrient cycling, and ensuring soil function (Makarov, 2019; Lei et al., 2022). A previous study by Liang et al. (2023) revealed a positive interaction between N-fixing bacteria and AMF. Furthermore, the coexistence of AMF and N-fixing bacteria synergistically enhances their individual effectiveness and positively contributes to the recovery and restoration of saline–alkali soil (Giambalvo et al., 2022). Within the microbial community, metabolic groups play a crucial role in sustaining bacterial growth by obtaining energy, vitamins, and carbohydrates from the soil (Srour et al., 2020). In this study, metabolism was found to be the prominent module among the six categories, which was consistent with the result of Pii et al. (2016). This result could be explained by the fact that modified materials contributed to an increase in soil nutrients, particularly organic matter, providing bacteria with an abundant source of metabolic substrates, thereby leading to significant alterations in the metabolic functions of the bacterial community (Liu et al., 2022).

## 5 Conclusion

Taken together, our study demonstrated that the application of modified materials effectively decreased soil salinity and increased soil nutrients (AP, AK, AHN, and SOM) and reshaped the microbial community composition, especially with regard to fungi. Specifically, modified materials significantly increased fungi diversity while reducing its richness, and at the phylum level, FA, SA, and TA notably increased the abundance of Ascomycota, while DE significantly augmented the abundance of Mortierellomycota. Additionally, the fungal biomarkers sensitive to modified materials were higher than the bacterial community. However, modified materials had a relatively minor impact on the bacterial community, only leading to changes in the bacterial community structure. Fungi exhibited a strong association with soil nutrients, while bacteria displayed insensitivity to soil nutrients. Overall, the effects of modified materials on soil nutrients and microbial communities varied depending on the changes in soil salinity caused by applying various modified materials. These results provide information to understand the relationships between soil properties and microbial communities, the restoration of soil in response to various modified materials, and insight into the restoration of saline–alkali land of Hetao Plain.

## Data availability statement

The data presented in the study are deposited in the NCBI Sequence Read Archive (SRA) database (Accession number: SRP471127).

## Author contributions

XB: Data curation, Formal analysis, Investigation, Methodology, Software, Writing – original draft, Writing – review & editing. EZ: Investigation, Supervision, Writing – review & editing. JW: Investigation, Project administration, Writing – review & editing. DM: Investigation, Writing – review & editing, Formal analysis, Methodology. CZ: Investigation, Writing – review & editing. BZ: Writing – review & editing. YL: Investigation, Writing – review & editing. ZZ: Investigation, Writing – review & editing, Software. FT: Conceptualization, Formal analysis, Investigation, Writing – review & editing, Resources. HZ: Investigation, Writing – review & editing. BW: Conceptualization, Funding acquisition, Investigation, Resources, Supervision, Writing – original draft, Formal analysis, Project administration, Visualization.
