# Supplementary material for: Soil fungal community is more sensitive than bacterial community to modified materials application in saline–alkali land of Hetao Plain
Source: Front Microbiol. 2024 Feb 5;15:1255536. doi: 10.3389/fmicb.2024.1255536 (PMC10875129; doi:10.3389/fmicb.2024.1255536)
Supplement: Supplementary file 1 [file Data_Sheet_1.docx]

Supplementary Material


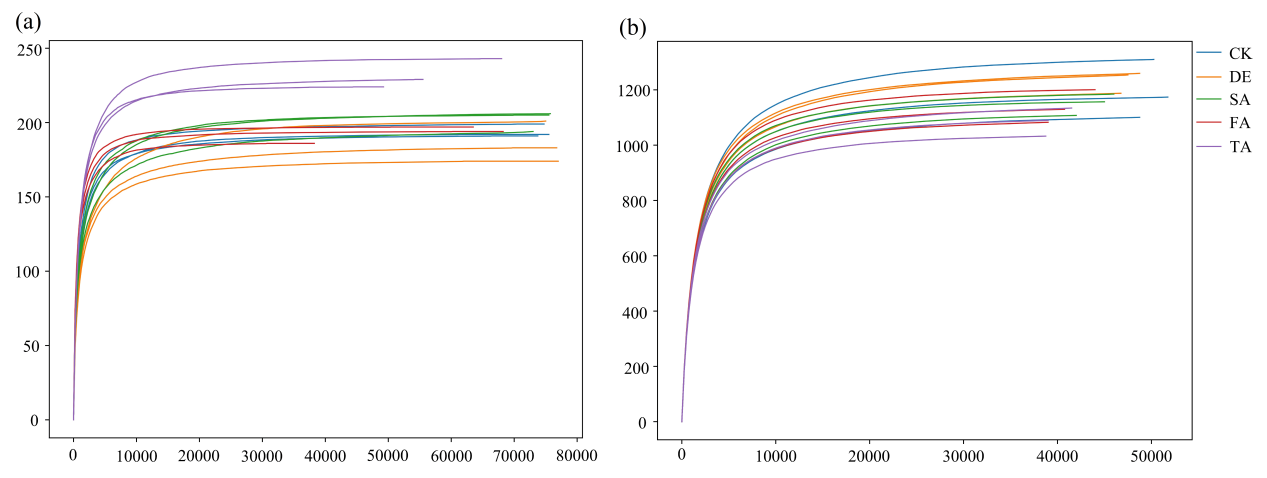


**Figure S1 The rarefaction curves of fungal (a) and bacterial (b) communities based on observed OTUs for individual soil sample.**


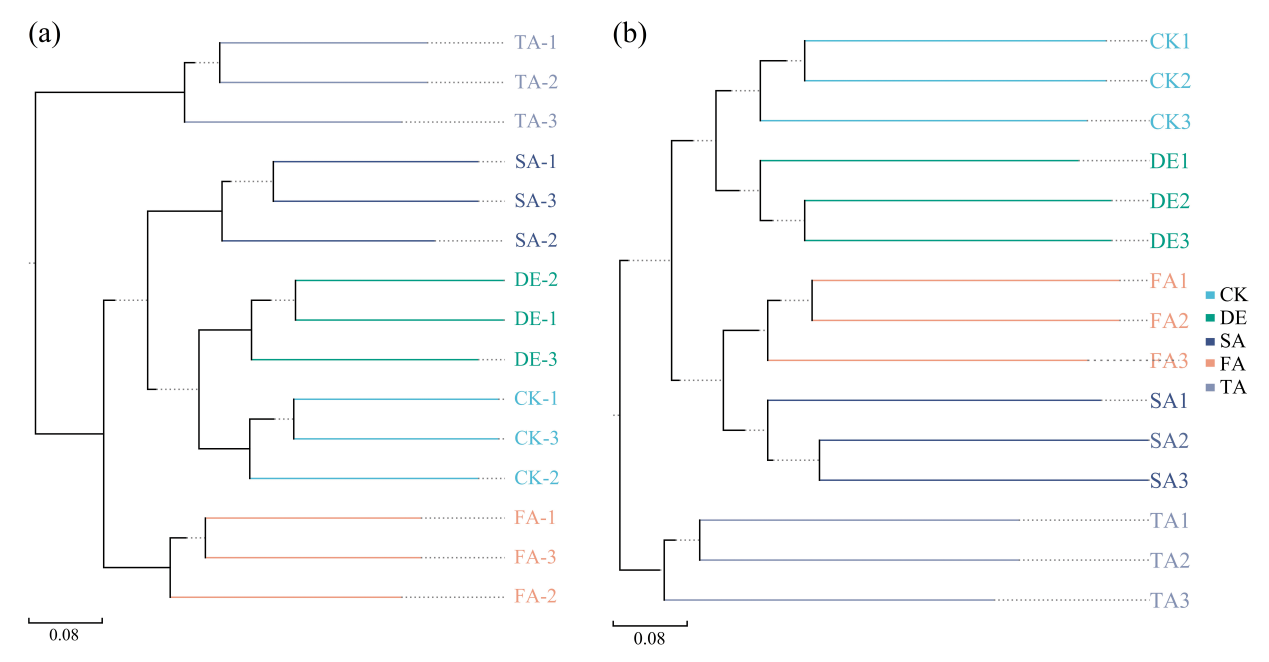


**Figure S2 UPGMA tree of fungal (a) and bacterial (b) community structures at the OTU level under modified materials**


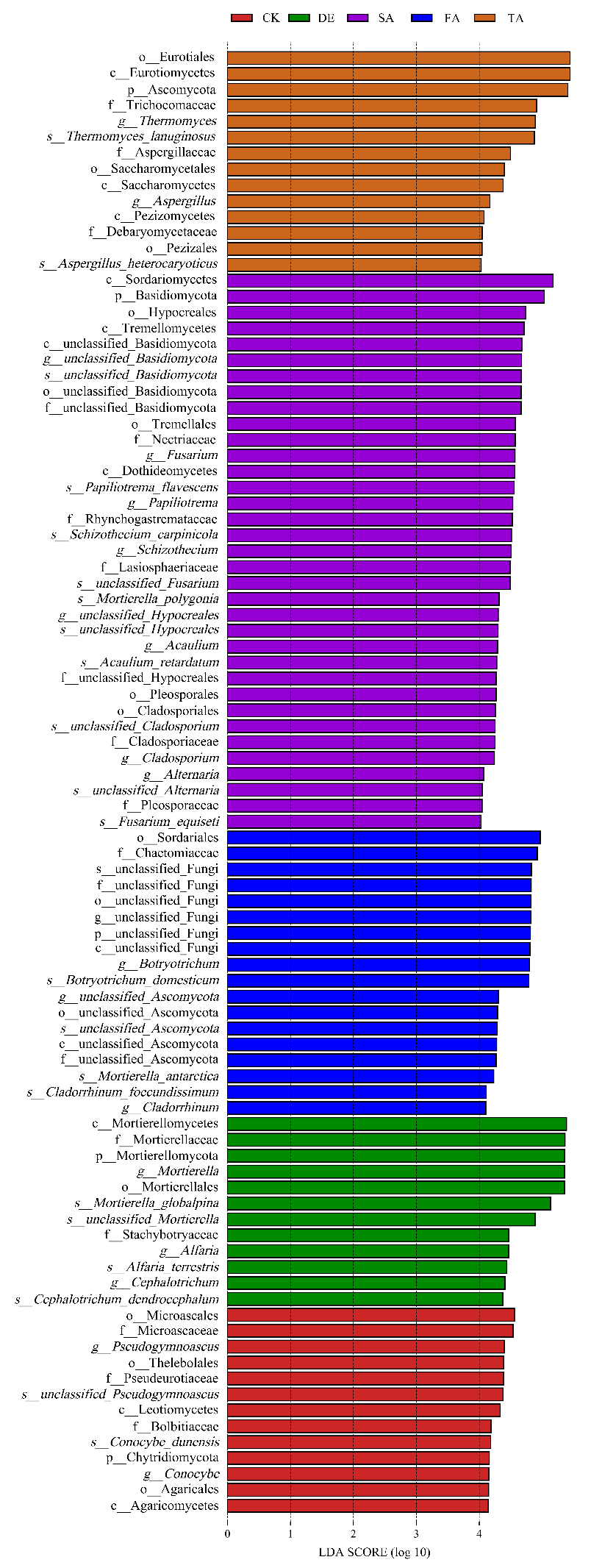


**Figure S3 Histogram of the linear discriminant analysis (LDA) scores of fungal communities computed for features differentially abundant among different modified materials (LDA score > 4).**


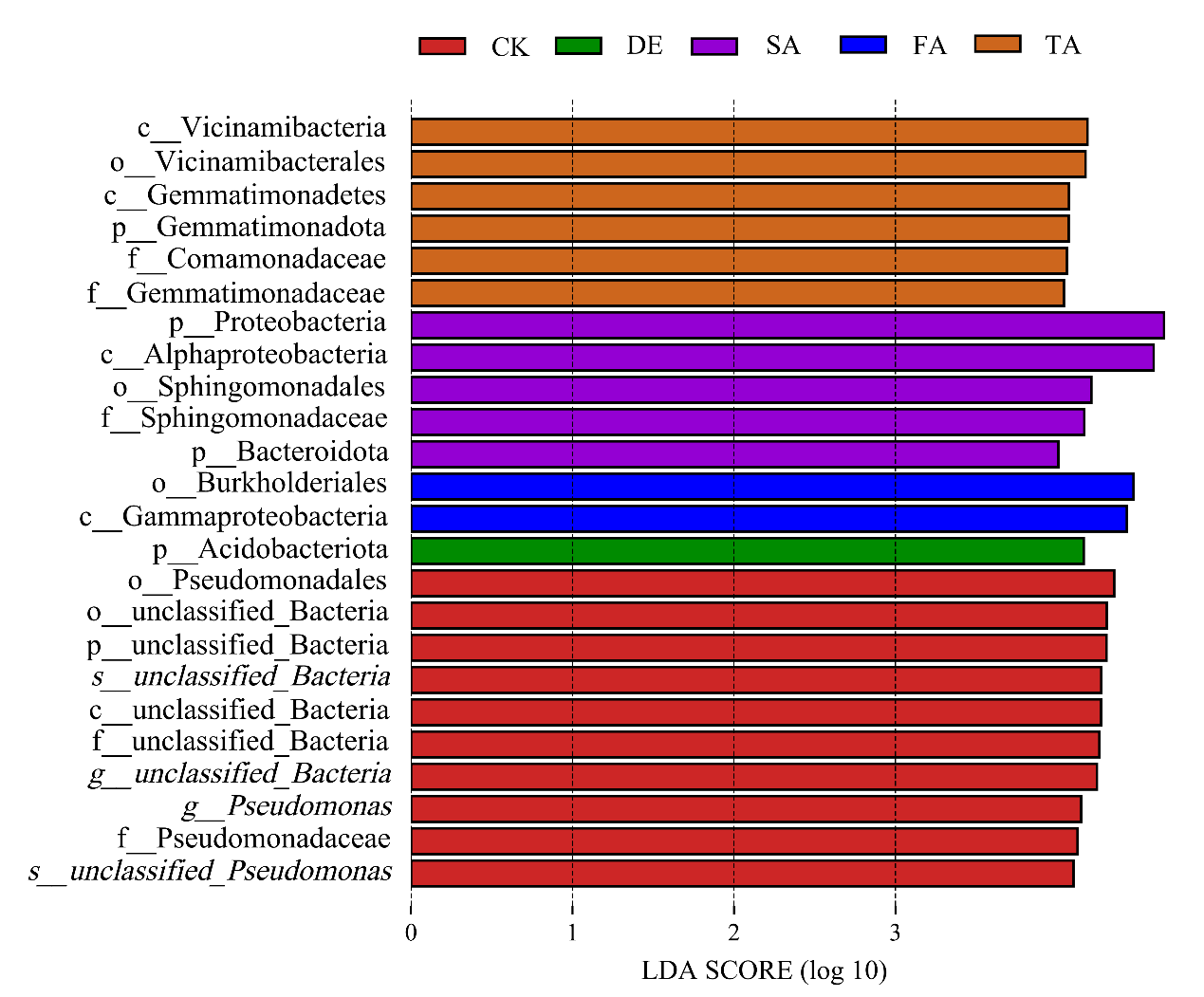


**Figure S4 Histogram of the linear discriminant analysis (LDA) scores of bacterial communities computed for features differentially abundant among different modified materials (LDA score > 4).**


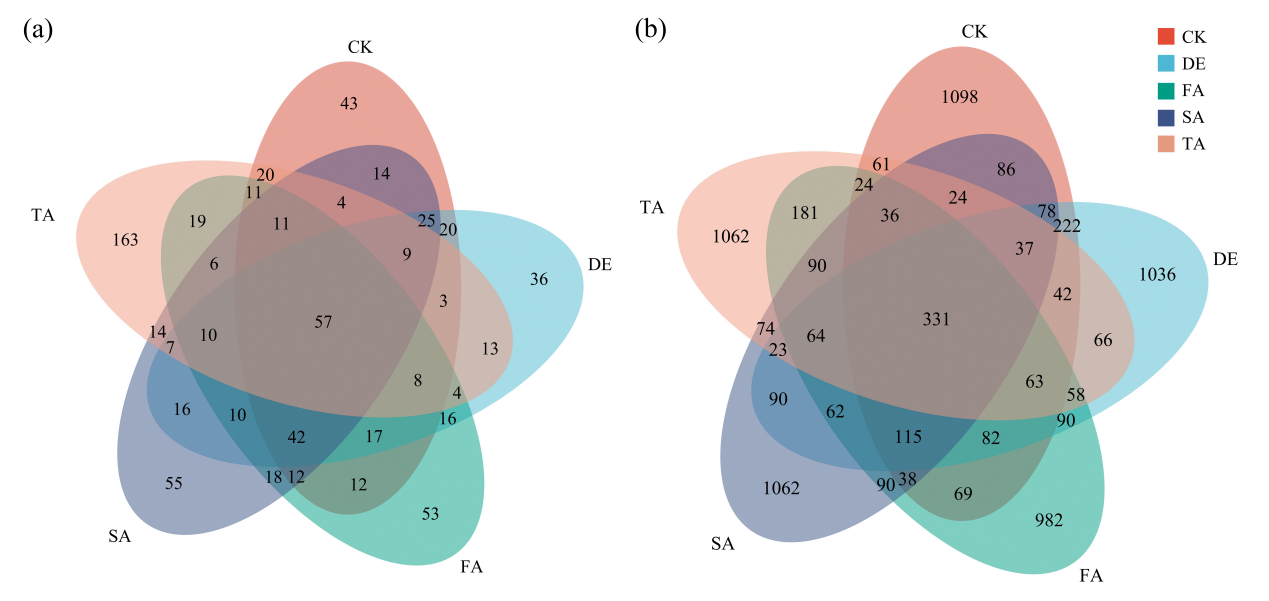


**Figure S5 Venn Diagram showing unique and common OTUs of fungi (a) and bacteria (b) under different modified treatments.**





**Figure S6 Bacterial functional predictions under modified materials annotated by PICRUSt2. a: level 1 function categories; b: level 2 function categories (relative abundance >1%).**
